# Polyethylene Glycol-grafted poly alpha-lipoic acid-dexamethasone nanoparticles for osteoarthritis

**DOI:** 10.3389/fddev.2023.1168287

**Published:** 2023-07-20

**Authors:** Yuanqiang Cheng, Zheng Jing, Yan Xu, Lihui Sun, Dongbo Li, Jianguo Liu, Dongsong Li

**Affiliations:** Department of Orthopaedics, First Affiliated Hospital of Jilin University, Changchun, China

**Keywords:** polylipoic acid, osteoarthritis, dexamethasone, nanoparticles, intraarticular injection, intraarticular injection of knee joint

## Abstract

Osteoarthritis (OA) is a chronic inflammatory disease that causes synovial hyperplasia, cartilage destruction, and the formation of bone spurs. Macrophages play an indispensable role in the pathogenesis of OA by producing proinflammatory cytokines. To achieve the effect of arthritis, hormones can effectively inhibit the progression of inflammation by inhibiting the secretion of inflammatory cytokines by macrophages in traditional therapy. However, the drug is quickly cleared from the joint space, and the high injection site infection rate and low local drug concentration make the clinical efficacy of corticosteroids greatly reduced. We described the design and preparation of Polyethylene Glycol-grafted Poly Alpha-lipoic Acid-dexamethasone Nanoparticles (NP_DXM/PPLA_), elucidated the mechanism of action of NP_DXM/PPLA_ in the treatment of OA in mice, and provided an experimental basis for investigating the treatment of OA with polymer nanoparticles loaded with dexamethasone. Flow cytometry and confocal laser scanning microscopy were used to confirm that NP_DXM/PPLA_ was well absorbed and released by macrophages, and it was discovered that NP_DXM/PPLA_ could efficiently reduce the proliferation of activated macrophages (RAW 264.7 cells). Enzyme-linked immunosorbent assay revealed that NP_DXM/PPLA_ could efficiently reduce the expression of proinflammatory cytokines IL-1β, IL-6, and TNF-α. The knee bone structure of OA mice was investigated by MicroCT, and it was discovered that intraarticular injection of NP_DXM/PPLA_ effectively alleviated the bone damage of the articular cartilage. Therefore, NP_DXM/PPLA_ is a potential therapeutic nanomedicine for the treatment of OA.

## 1 Introduction

Osteoarthritis is commonly an age-related disease with a degenerative illness of the joints. The most common symptoms of osteoarthritis are articular cartilage wear and synovitis, which causes pain and swelling in the joint, as well as limited activity (French et al., 2013; Fernanda et al., 2018). OA affects not only articular cartilage but also the entire joint, including the subchondral bone, ligament, synovium, meniscus, and even the muscles around the joint (Martel-Pelletier et al., 2012). Although the pathogenesis of OA is not fully understood, the disease is characterized by the gradual degeneration of articular cartilage. Recent studies found that this progressive degeneration is related to oxidative stress, and reactive oxygen species play a significant role in this procedure (Lepetsos and Papavassiliou, 2016). At the same time, the inflammatory microenvironment plays a significant role in the occurrence and development of OA. The severe inflammatory response of macrophages results in the recruitment of a large number of inflammatory cells and the secretion of high levels of proinflammatory cytokines such as IL-1β, IL-6, TNF-A, and matrix metalloproteinases (MMPs) at the lesion site. Reduces proteoglycan synthesis and type II collagen, and aggravates cartilage erosion and degeneration (Bondeson et al., 2006; Blom et al., 2007; Crielaard et al., 2012; Sonderegger et al., 2012; Agarwal et al., 2016; Sang et al., 2016).

In clinical practice, intraarticular injection of corticosteroids or hyaluronic acid is often employed to relieve pain and control inflammation (Jones et al., 2018; Conaghan et al., 2019). Although corticosteroids can alleviate pain and other symptoms, their related side effects and the rate of joint cavity clearance severely limit their clinical application (Evans et al., 2014; Brown et al., 2019). Corticosteroids injected into the joint cavity are cleared with a half-life of 1–4 h (Brown et al., 2019). Multiple intraarticular injections are needed to achieve therapeutic effects. However, repeated intraarticular injections can cause joint infection, and long-term corticosteroids use can destroy articular cartilage and hasten joint degeneration (McAlindon et al., 2018). With the continuous development of medical chemistry, researchers have used nanomaterials as drug delivery carriers. Nanomaterials have consistently demonstrated improved drug retention properties in the joint cavity and drug delivery to the joint when compared to free drug injection. Furthermore, active and passive targeting strategies can be used to modify nanomaterials to promote interaction and localization with specific articular tissues such as cartilage and synovium (Brown et al., 2019). In addition, *α*-lipoic acid (αLA) is a natural antioxidant synthesized in the human body and an important cofactor of mitochondrial metabolism, which has been employed in the treatment of Alzheimer’s illness and diabetes (Maczurek et al., 2008; Singh and Jialal, 2008; Solmonson and Deberardinis, 2017). It has been discovered that by heating above its melting point, αLA can be polymerized to poly *α*-lipoic acid (PαLA) without the use of a catalyst or solvent. PαLA is a drug carrier with great potential for development. PαLA and its degradation products are safe and biocompatible. Disulfide bonds in its main chain play an antioxidant role in osteoarthritis (Packer et al., 1995; Shimoda et al., 2007; Shay et al., 2009; Li et al., 2013; Yang et al., 2018). Given the use of nanomaterials as corticosteroids carriers and PLA’s excellent anti-inflammatory properties, we speculated that NP_DXM/PPLA_ prepared by the electrostatic and hydrophobic action of the carboxyl group on mPEG-g-PαLA carrying DXM may be a promising new drug for the treatment of OA.

**FIGURE 1:**
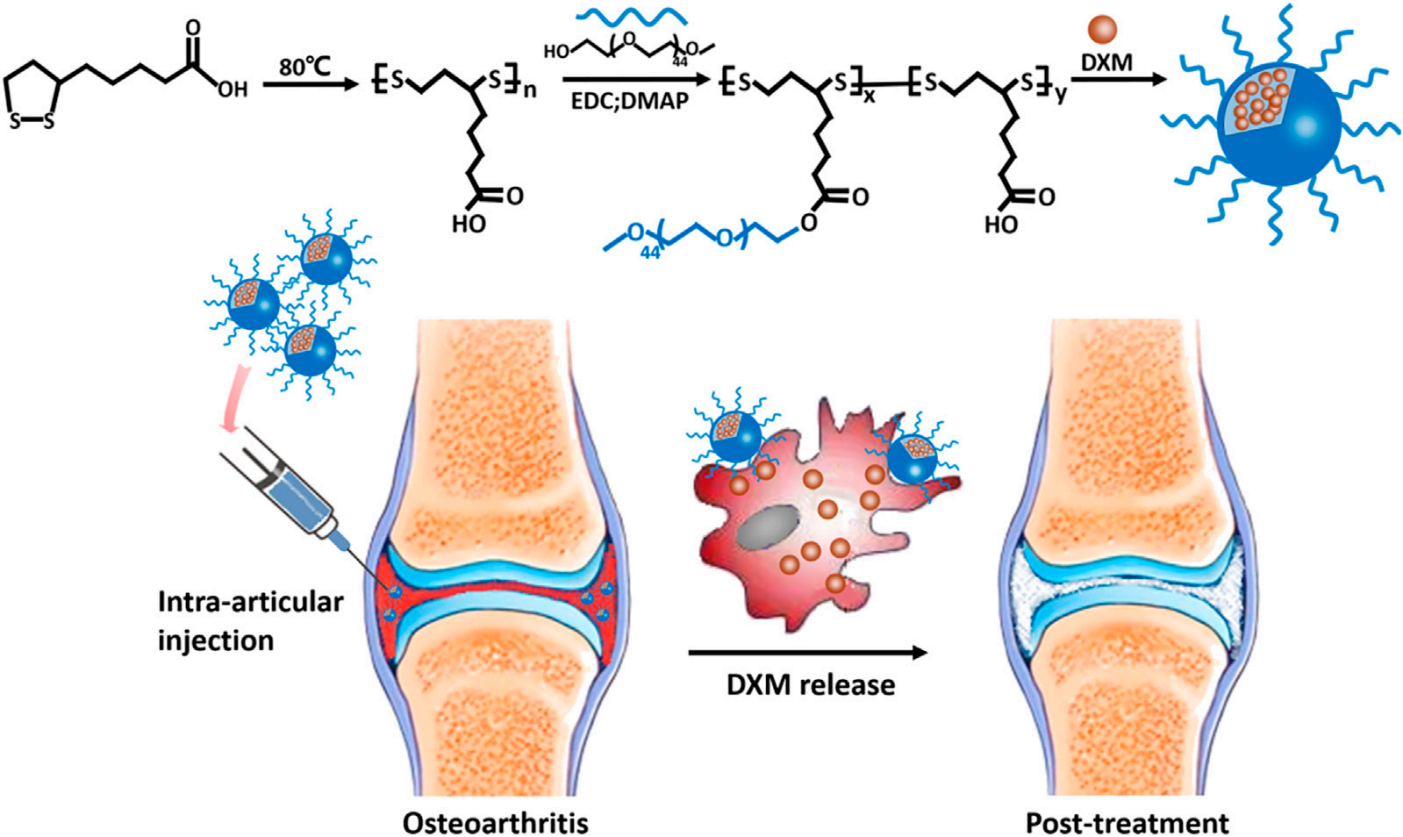
Schematic illustration of the preparation of NP_DXM/PPLA_ NPs for treatment of OA.

To test this hypothesis, we created and tested an NP_DXM/PPLA_ for the effective treatment of OA (Figure 1.). NP_DXM/PPLA_ was prepared from αLA by the polymerization reaction. The hydrophilic polymer mPEG was electrostatically linked to the NP_DXM/PPLA_ by the hydrophobic DXM of the carboxyl group on mPEG-g-PαLA. The anti-inflammatory effects of NP_DXM/PPLA_ nanoparticles on OA mice induced by monosodium iodoacetate (MIA) were investigated further. The outcome revealed that NP_DXM/PPLA_ could effectively carry DXM and had a better effect on OA treatment than traditional DXM injection alone.

## 2 Materials and methods

### 2.1 Materials

Dexamethasone (DXM) (average molecular weight Mn = 206.362 g mol^−1^), Poly (ethylene glycol) monomethyl ether (mPEG), *α*-lipoic acid (average molecular weight Mn = 2000 g mol^−1^), 4′, 6-diamidine-2-phenylindole (DAPI), 4-dimethylamino pyridine (DMAP), 3-(4, 5-dimethyl-thiazole-2-yl)-2, 5-diphenyltetrazolium bromide (MTT), Sigma–Aldrich (Sigma, Germany) provided the 1-(3-(dimethylamino) propyl) -3-ethylcarbondiimine hydrochloride (EDCHCl), and sodium iodoacetate (MIA). Thermo Fisher Scientific (China) Co., Ltd. provided cell culture substrates such as Dulbecco’s modified Eagle’s medium (DMEM) and fetal bovine serum (FBS) (Shanghai, China). Penicillin and streptomycin were purchased from Shijiazhuang Huabei Pharmaceutical Co., Ltd. Biosharp Co., LTD. provided the lipopolysaccharide (LPS). Sinopharm Chemical Reagent Co., LTD. supplied N, N-dimethylformamide (DMF), and Sinopharm Chemical Reagent Co., LTD., supplied tetrahydrofuran (THF). All other chemicals were purchased commercially.

### 2.2 Preparation and characterization of mPEG-g-PαLA (NP_PPLA_) and NP_DXM/PPLA_

#### 2.2.1 The synthesis of mPEG-g-PαLA

First, PαLA was prepared by a reaction between αLA with diethyl ether. Then, 1.68 g PαLA and 4.0 g mPEG were dissolved in 50 mL Tetrahydrofuran (THF) and 50 mL dimethyl sulfoxide (DMSO), respectively. Then mixed the two solutions slowly with a pipette gun, then 333.4 mg EDCHCl and 56.09 mg DMAP were added. After 24 h of stirring at room temperature, adding the same catalyst to the reaction solution, and continuing the reaction for another 24 h, the mixed solution was dialyzed in Milli-Q water [molecular weight cut-off (MWCO) = 3,500 Da], the dialysis time was 5 days, and the deionized water was changed 3 times per day. Also, the mPEG-g-PαLA was obtained by lyophilization (Scientz-12ND lyophilizer, Ningbo Scientz Equipment limited by share Ltd., China). Bruker AV 300 NMR spectrometer (AVANCE III, Bruker corporation, Switzerland) was used to measure the proton nuclear magnetic resonance (1H NMR) spectra of PαLA and mPEG-g-PαLA.

#### 2.2.2 The synthesis of mPEG-g-PαLA (NP_PPLA_)and NP_DXM/PPLA_

First, 100 mg mPEG-g-PαLA was dissolved in 8 mL DMSO, then dropped into 100 mL deionized water and stirred for 2 h to prepare mPEG-g-PαLA. The solution was then transferred to an MWCO 3500 dialysis bag for 10 h to remove DMSO before being lyophilized to obtain NP_PPLA_. Again, 100 mg mPEG-g-PαLA and 20 mg DXM were dissolved in 8 mL DMSO, dropped into 100 mL deionized water, stirred for 2 h, then transferred to the MWCO 3500 dialysis bag for 10 h to remove DMSO, and NP_DXM/PPLA_ NPs were lyophilized. The Dio-labeled NP_PPLA_ were prepared following the same method and were denoted as NP_Dio/PPLA_.

Transmission electron microscopy (TEM) and dynamic laser scattering were used to determine the hydrodynamic radius (Rh) of NPDXM/PPLA NPs (DLS). TEM test: First, weigh 2 mg NP_PPLA_ and NP_DXM/PPLA_ respectively, and configure them into 0.1 mg/mL solution. The solution should then be pipetted onto a clean copper net with a 20 μL pipette and allowed to dry at room temperature for 24 h. TEM was also performed using a JEOL JEM-1011 transmission electron microscope (JEOL, Ltd., Tokyo, Japan) with a 100 kV accelerating voltage. DLS test: First, weigh 2 mg NP_PPLA_ and NP_DXM/PPLA_, respectively, and dissolved them in PBS solution with pH 7.4 for later use. The Wyatt QELS instrument was then set up to measure the fluid dynamic radius (Rh) of the NP_PPLA_ and NP_DXM/PPLA._

### 2.3 *In vitro* release of DXM from NP_DXM/PPLA_

*In vitro* DXM release of NP_DXM/PPLA_ was evaluated in PBS containing 200 U mL^−1^ esterase at pH = 7.4. In short, NP_DXM/PPLA_ is released in PBS buffers with or without H_2_O_2_. To begin, 3.0 mg NP_DXM/PPLA_ was weighed and dissolved in 15 mL pH= 7.4 PBS buffer before being transferred to an MWCO 3500 dialysis bag for standby. The solution should then be added to a different medium every 200 mL. Medium (I) was 100 mL PBS buffer with pH = 7.4; Medium (II) was 100 mL PBS buffer containing 10 mol H_2_O_2_ with pH = 7.4; Medium (III) was 100 mL PBS buffer containing 1 mol H_2_O_2_ with pH = 7.4. The dialysis bags were then placed in beakers containing various release media and placed in a 37°C constant temperature oscillating box that was continuously shaking at 100 RPM to simulate the human body environment. The release solution in the 2.0 mL beaker was removed with a pipetting gun at a predetermined time point, and 2.0 mL of the media in the beaker was added. The content of DXM was determined by high-performance liquid chromatography (HPLC) (Flexar, PerkinElmer, Shelton, United States of America). The mobile phase was methanol-water (60:40, V/V) at a flow rate of 1.0 mL min^−1^ in an analytical C18 column (5 m, 250 4.6 mm, PerkinElmer Brownlee, United States). The detection wavelength was 240 nm, and the column temperature was 25°C. The injection volume was 20 μL. The limits of detection and quantitation were 26.2 and 87.2 ng mL^−1^, respectively. The calibration curve was linear in the range of 110–7,780 ng mL^−1^ (r2 = 0.999). The release curve was drawn using the standard curve method, which is used to calculate the DXM concentration released at each time point.

### 2.4 Determination of the stability of NP_DXM/PPLA_

NP_DXM/PPLA_ stability test: 0.5 mg NP_DXM/PPLA_ was dissolved in 5 mL PBS with pH = 7.4 to prepare 0.1 mg/mL solution. The solution was then transferred to the DLS sample bottle, which was oscillated in a 37°C constant temperature oscillating chamber at a constant temperature of 100 rpm to simulate the internal environment of the human body. The change in particle size was detected and plotted by the Wyatt QELS device at specified time points.

### 2.5 Endocytosis and release of NP_Dio/PPLA_ from RAW 264.7 cells monitored by FCM and CLSM

Entosis and the release of NPDio/PPLA from RAW 264.7 cells were detected using confocal laser scanning microscopy (CLSM) and flow cytometry. In brief, RAW 264.7 cells were seeded into a six-well plate at a density of 5 × 104 cells per well, add 2 mL DMEM medium containing 10% (V/V) FBS and 1% (W/V) penicillin-streptomycin, and cultured for 12 h in a 5% carbon dioxide (CO_2_) incubator at 37 °C in a humid environment. Then, RAW 264.7 cells were activated with 1 μg ML^−1^ LPS for 24 h. Following activation of the macrophages, the medium was replaced with DMEM medium containing NP_Dio/PPLA_ at a Dio concentration of 1 μg ML^−1^. Then, the medium was incubated at 37 °C for 0.5 h, 2 h, 4 h, 6 h, 8 h, and 10 h. Following that, the medium was removed, the cells were washed three times with PBS, fixed with 4% paraformaldehyde, and the nuclei stained with 4’,6-diamidine-2-phenylindole (DAPI) Aladdin. Finally, using an LSM 543 CLSM microscope (Carl Zeiss, Jena, Germany) with a 20-eyepiece objective lens, microscopic images of cell uptake were obtained. For FCM analysis, activated RAW 264.7 cells were co-cultured with NP_Dio/PPLA_. Following that, the medium was removed, and the cells were washed three times with PBS, digested with trypsin, and centrifuged for 5 minutes to collect the cells. Finally, they were suspended in 500 μL PBS, and the fluorescence intensity of cell uptake was measured using a Guava EasyCyte 12 flow cytometer (Millipore, Billerica, MA, United States).

### 2.6 Cell viability assay

The toxicity of NP_PPLA_, NP_DXM/PPLA,_ and free DXM to RAW 264.7 cells was determined using MTT. In brief, RAW 264.7 cells were sown into 96-well plates at a density of 7,000 cells per well. Following a 24-h culture with or without LPS (1 μg mL^−1^), the medium was replaced with 200 μL of DMEM medium containing free DXM, NP_PPLA_, and NP_DXM/ PPLA_. The concentration in the medium containing DXM was from 0.048 to 50 μg mL^−1^. After 24 h, add 20 μL of 5% MTT solution and incubate for another 4 h. Following that, 150.0 μL DMSO was added to the medium, and the absorbance of each well was measured at 490 nm using a multifunctional microplate reader (Spark, TECAN, Switzerland).

### 2.7 Expression of proinflammatory cytokines

The expression of proinflammatory cytokines were determined by enzyme-linked immunosorbent assay (ELISA) in activated RAW 264.7 cells. In brief, the cells were seeded at a density of 4 105 cells/well in 6-well plates and cultured for 24 h. RAW 264. For 24 h, 7 cells were activated with 1 μg mL^−1^ LPS. Following that, 2 mL of DMEM containing saline, NP_PPLA_, NP_DXM/PPLA,_ or free DXM at a final TA concentration of 1 μg mL^−1^ was added to the cell culture medium. After 24 h, IL-1β, TNF-α, and IL-6 levels in the supernatants were determined using ELISA kits (Spark^®^ multimode microplate reader, TECAN, Switzerland).

### 2.8 *In vivo* therapeutic effect of NPDXM/PPLA on OA mice

50 BALB/c mice (six to eight weeks old) were purchased from the Chinese Academy of Medical Sciences Institute of Experimental Animals. All animal procedures were following the “Jilin University Laboratory Animal Care” and Use Guidelines verified by the Animal Ethics Committee of the First Hospital of Jilin University. First, 40 mice were given general anesthesia with pentobarbital sodium (2%). To induce OA, MIA (5 mg kg^−1^, Sigma–Aldrich, st. Louis, MO, United States of America) was injected intraarticular through the subpatellar ligament of the left knee with a 30G needle (5 mg kg^−1^, Sigma–Aldrich, st. Louis, MO, United States of America) in mice (Chung et al., 2015; Sun et al., 2018). The successful induction of OA was confirmed by significantly reducing the load-bearing and withdrawal point stimulus thresholds of the hind paw (Bove et al., 2003; Nwosu et al., 2016). Mice without OA induction were used as a negative control.

Three days after OA induction, the mice were randomly divided into four groups and given different treatments: normal saline, DXM, NP_PPLA_, and NP_DXM/PPLA_, respectively. The OA knee was tested by intraarticular injection in each group. The preparations concentration in the DXM, NP_PPLA,_ and NP_DXM/PPLA_ groups remained constant at 1.0 mg kg^−1^. Day zero was the first day of treatment. All groups were given treatments every 4 days until the mice were put down at the end of the third week.

The knee joint was harvested and treated for further analysis after the animals were killed. First, the knee joint specimens of mice were fixed in 10% formalin buffer at 4 °C for 24 h and then decalcified with EDTA for 1 h. Next, the decalcified specimens were buried in paraffin and cut into 5 μm sections. Immunohistochemistry was used to stain sections with hematoxylin and eosin (H&E), IL-1, TNF-, and IL-6 (Affinity Biosciences, OH, United States of America). Knee specimens were also used to assess the shape of the knee and to measure skeletal characteristics with Micro CT.

### 2.9 Statistical analysis

All results are presented as the means ± standard deviations, and the important statistical data were analyzed using one-way ANOVA in the GraphPad Prism Software (GraphPad Software Inc., San Diego, United States of America).* indicates that *p* < 0.05 was considered statistically significant.; ** represents 0.01 < *p* < 0.05 and *** represents *p* < 0.001 was considered to be more highly statistically significant.

## 3 Results and discussion

### 3.1 Preparation and characterization of mPEG-g-PαLA

**FIGURE 2:**
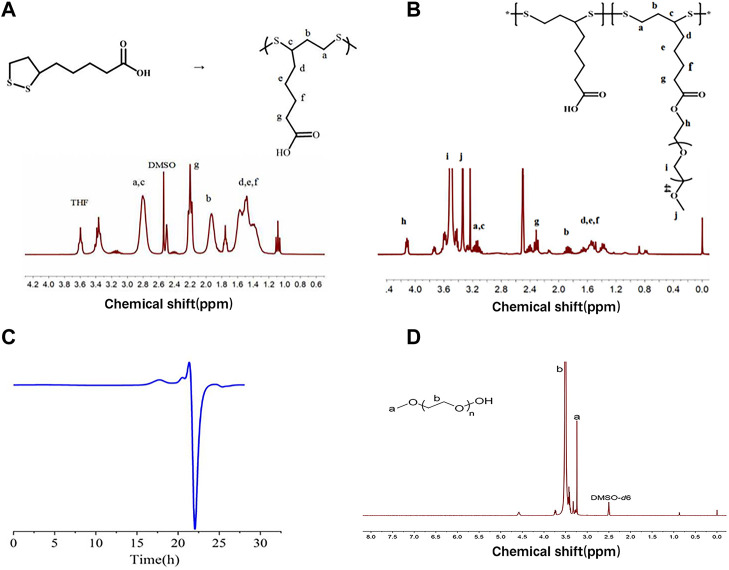
**(A)** Polymerization of αLA and ^1^H NMR spectra of PαLA (DMSO-D6) **(B)** ^1^H NMR spectra of PαLA (DMSO-D6) of mPEG-g-PαLA (DMSO). **(C)** GPC analysis of DMF phase of mPEG-g-PαLA.

As shown in Figure 2A, PαLA was synthesized by ring-opening polymerization of the aLA monomer at 80°C. The structure of PαLA was confirmed by ^1^H NMR. The mPEG-g-PαLA polymer was created by esterifying the hydroxyl group of mPEG with the carboxyl group of PαLA and coupling the mPEG with the PαLA side chain. 1H NMR spectroscopy confirmed the structure of the obtained mPEG-g-PαLA. As shown in Figure 2B, the positions of the proton peaks H, I, and J of polymer mPEG-g-PαLA in the ^1^H NMR spectrum indicate that mPEG has successfully connected with the P_αLA_ side group. Furthermore, software integration of H and B peaks revealed that mPEG modified 50% of the carboxyl groups on the PαLA side chain. In addition, the structure of the product was analyzed by gel permeation chromatography (GPC). The peak time of GPC in Figure 2C is 17.9 min, indicating the weight of mPEG-g-PαLA is Mw = 7.76 × 10^4^ Da, proving that mPEG-g-PαLA has been successfully grafted. In summary, this means that mPEG successfully connects to the side chain of PαLA.

### 3.2 Preparation and characterization of NP_PPLA_ and NP_DXM/PPLA_

**FIGURE 3:**
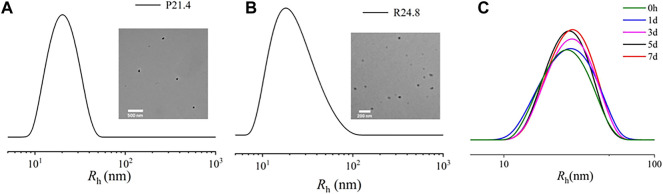
**(A)** Sizes of NP_PPLA_ measured by DLS and TEM images of NP_PPLA_. **(B)** Sizes of NP_DXM/PPLA_ measured by DLS and TEM images of NP_DXM/PPLA_. **(C)** The particle size of NP_DXM/PPLA_ at different times tested by DLS.

The Rh of NP_PPLA_ measured by DLS was (21.4 ± 1.7) nm, and TEM images showed an irregular shape of NP_PPLA_ (Figure 3A). Meanwhile, the Rh and TEM of NP_DXM/PPLA_ were also tested. NP_DXM/PPLA_ had an Rh of (24.0 ± 1.2) nm, and TEM images revealed a quasi-circular structure (Figure 3B). We found a slight increase in the size of NP_DXM/PPLA_, which also indicates that DXM is successfully encapsulated in nanoparticles. DXM’s drug loading content and drug loading efficiency were both as expected at 10.1% and 80.7%, respectively. Due to the amphiphilic properties of polymers, drug delivery efficiency is improved. Furthermore, DLS was used to test the particle size change of NP_DXM/PPLA_ at different times in PBS with pH = 7.4 (Figure 3C), and it was found that the particle size change of NP_DXM/PPLA_ was not obvious within 7 days, indicating that the nanoparticles were relatively stable under normal physiological state. And can maintain the shaped structure for a long time.

### 3.3 *In vitro* drug release of NP_DXM/PPLA_

**FIGURE 4:**
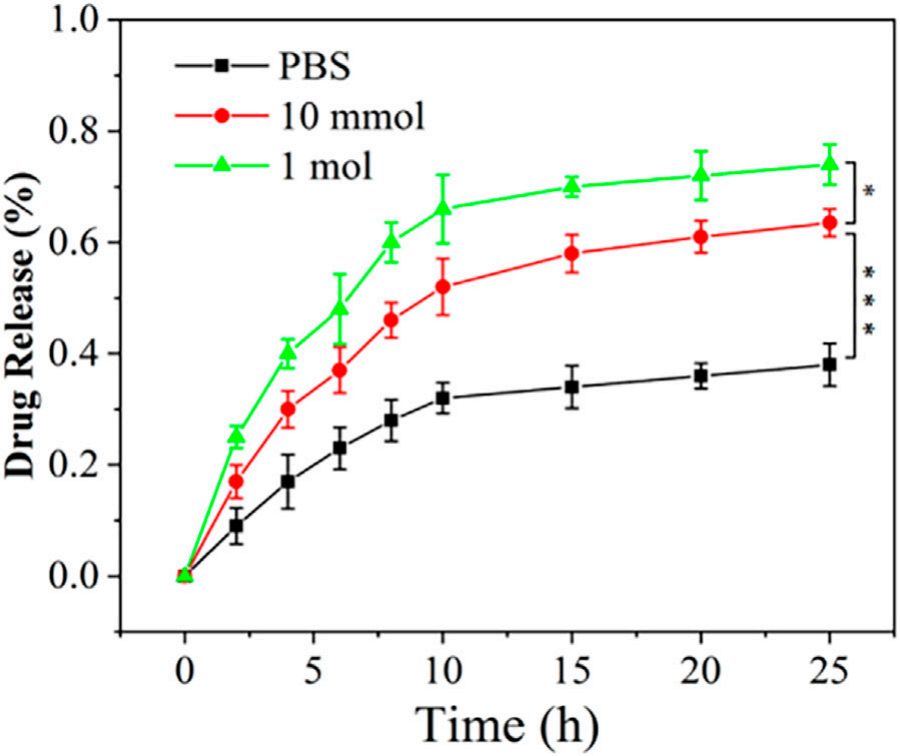
The release of DXM from NP_DXM/PPLA_ in PBS (pH 7.4) containing 200 U mL^−1^ Esterase.

During the development of OA, activated macrophages are relevant to the unregulated expression of matrix metalloproteinases (MMPs), proinflammatory cytokines, and other tissue-degrading enzymes (Bondeson et al., 2006; Bondeson et al., 2010; Sellam and Berenbaum, 2010). It has been reported that matrix metalloproteinases break ester bonds (Xia et al., 2001; Giannelli et al., 2004). As a result, detecting DXM release from NP_DXM/PPLA_ in PBS (pH 7.4, containing 200 U ML^−1^ esterase) can mimic DXM release in OA (Joshi et al., 2018). *In vitro* drug release of NP_DXM/PPLA_ was performed in PBS buffer with or without H_2_O_2_, and the release curve was determined by HPLC. Figure 4 depicts the results. When the pH is 7.4, we can divide the NP_DXM/PPLA_ release into three periods: (1) rapid release stage; DXM released in PBS at pH = 7.4, PBS containing 10 mmol H_2_O_2_ at pH = 7.4 and PBS containing 1 mol H_2_O_2_ at pH = 7.4 was 33%, 53%, and 68%, respectively, in the first 10 h (2) Slow-release stage: 38%, 62%, and 73% were released within 20 h (3) Plateau stage: DXM was rarely released. The amount of DXM released increased significantly as the concentration of H_2_O_2_ in the release medium increased. The increase in DXM release should be attributed to the H_2_O_2_-mediated cleavage of NP_DXM/PPLA_. As a result, we concluded that NP_DXM/PPLA_ can release DXM stably under physiological conditions, but can accelerate DXM release under the condition of higher oxidant concentration.

### 3.4 Endocytosis and intracellular drug release

**FIGURE 5:**
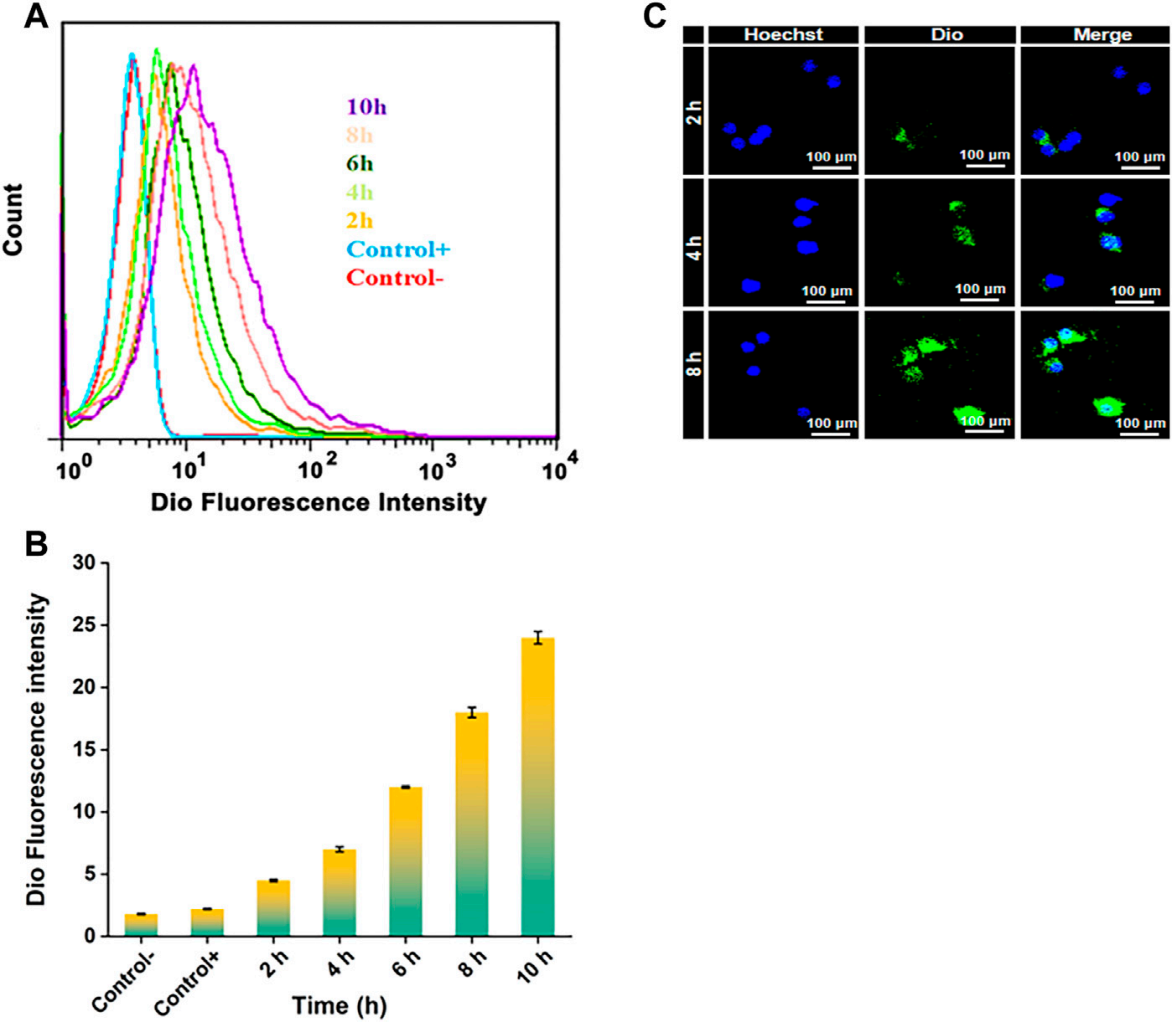
**(A)** FCA of RAW 264.7 cells: Cultured in DMEM medium containing NP_Dio/PPLA_ for 2 h, 4 h, 6 h, 8 h, 10 h with 1 μg mL^−1^ LPS. The average fluorescence intensity of Dio at each time point was detected. **(B)** FCA of RAW 264.7 cells: Cultured in DMEM medium containing NP_Dio/PPLA_ for 2 h, 4 h, 6 h, 8 h, 10 h without 1 μg mL^−1^ LPS. The average fluorescence intensity of Dio at each time point was detected **(C)** Confocal scanning microscope images of RAW 264.7 cells co-cultured with DMEM medium containing NP_Dio/PPLA_ with 1 μg mL^−1^ LPS for 2 h, 4 h, and 8 h.

The endocytosis of NPDio/PPLA to activated RAW 264.7 cells was studied using FCA and CLSM to demonstrate the endocytosis and intracellular drug release ability of activated RAW 264.7 cells to NP_Dio/PPLA_. When activated RAW 264.7 cells were treated with NP_Dio/PPLA_, the fluorescence intensity increased with increasing incubation time as shown in Figures 5A,B. In addition, the fluorescence intensity reached a stable state after 8 h of incubation, indicating that macrophages’ internalization of NP_Dio/PPLA_ nanoparticles reached saturation after 8 h. In contrast, activated RAW 264 showed an extremely low fluorescence signal. NP_PPLA_ or non-LPS-activated RAW 264. Was applied to 7 cells. Seven cell, implying that macrophages could release NP_Dio/PPLA_ nanoparticles could be released by macrophages. RAW 2647 cells were further examined by CLSM (Figure 5C). DAPI nuclei were blue and Dio fluorescence was green. Dio is a lipophilic membrane dye that can only be diffused laterally into cells, staining the entire cell membrane gradually. Green fluorescence surrounds blue fluorescence, indicating that the released Dio enters the cell, and green fluorescence intensity increases significantly over time. The results were consistent with the FCA results. In conclusion, NP_Dio/PPLA_ can be effectively endocytosis and released into macrophages by activated RAW 264.7 cells.

### 3.5 The ability of NP_DXM/PPLA_ to inhibit activated macrophages

**FIGURE 6:**
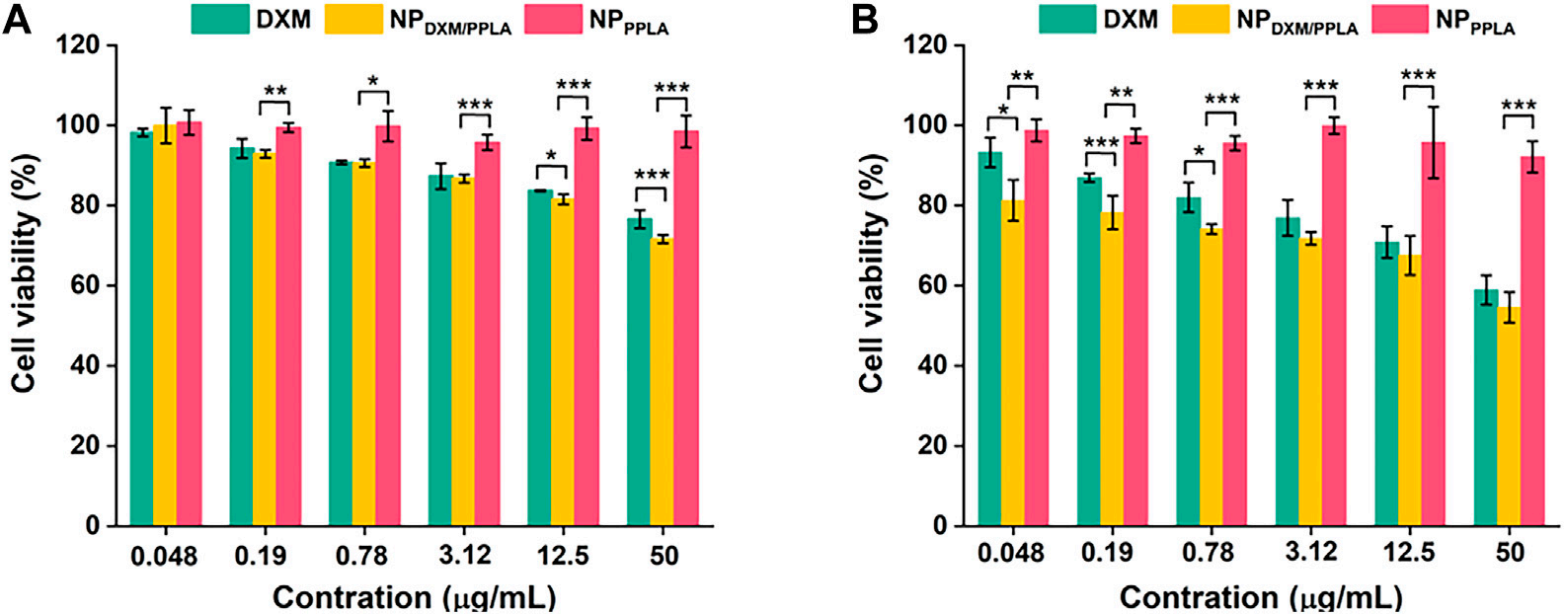
**(A)** Inhibitory effects of DXM, NP_PPLA_ and NP_DXM/PPLA_ on RAW 264.7 cells in the presence of 1 μg mL^−1^ LPS **(B)** Inhibitory effects of DXM, NP_PPLA_ and NP_DXM/PPLA_ on RAW 264.7 cells in the absence of 1 μg mL^−1^ LPS.

PPLA was non-toxic to RAW 264.7 cells and inhibited macrophage activity, as shown in Figure 6. αLA, a natural antioxidant synthesized in the human body, does not affect RAW 264.7 cells’ activity. In addition, the poor solubility of DXM may be responsible for the decreased inhibition of RAW 264.7 cells. In the absence of LPS, with the DXM concentration increasing to 50 μg mL^−1^, the RAW 264.7 cell activity of the NP_DXM/PPLA_ group was 70%, and cell inhibition was up to 30% compared to the DXM and NP_PPLA_ groups (Figure 6A). This may be due to the endocytosis of nanoparticles by macrophages. Furthermore, the cell inhibition effect of the NPDXM/PPLA group after LPS activation was 43%, indicating that the inhibition effect of NP_DXM/PPLA_ on the proliferation of activated RAW 264.7 cells increased (Figure 6B).

### 3.6 Therapeutic effect and expression of proinflammatory cytokines in OA mice

**FIGURE 7:**
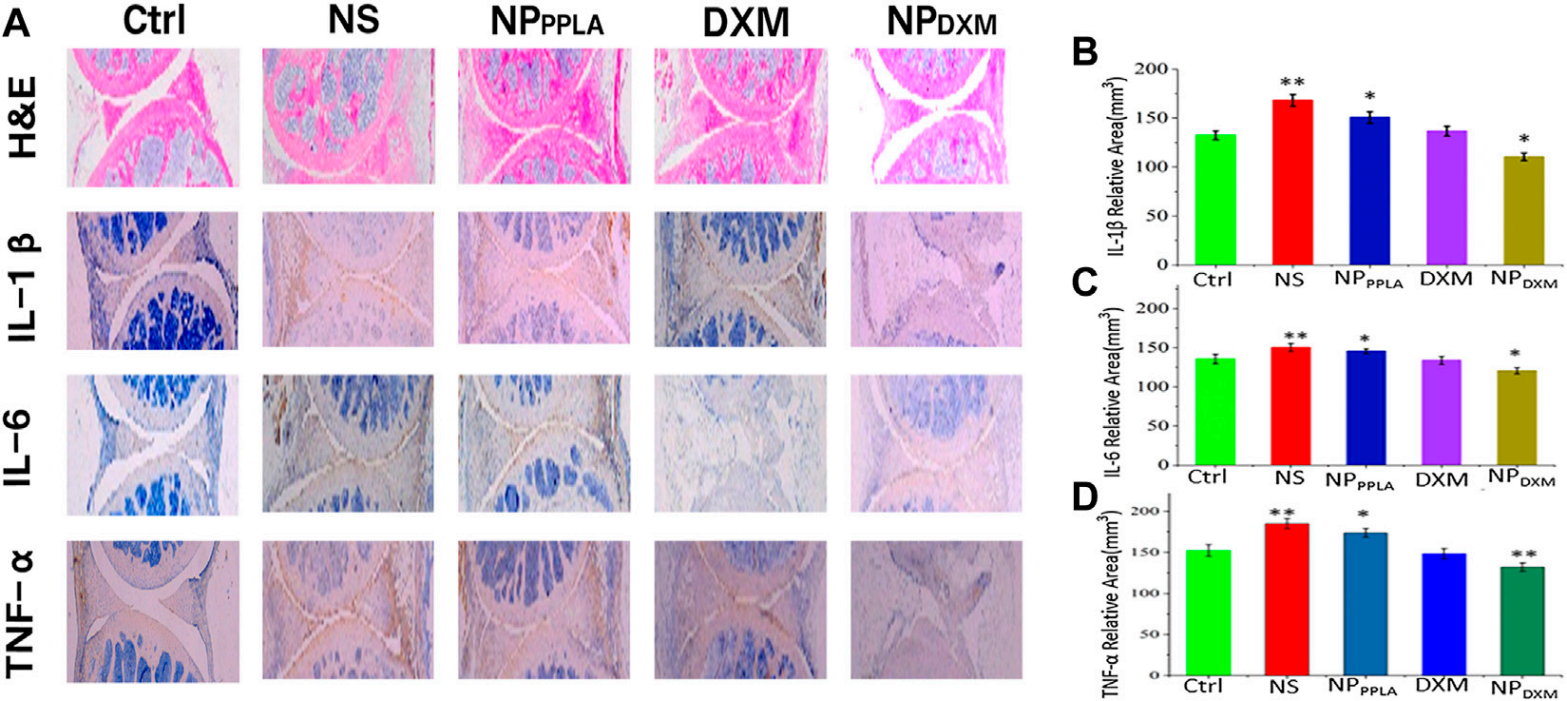
**(A)** The efficacies of NS, NP_PPLA_, DXM and NP_DXM/PPLA_ on OA were observed by H&E staining joint sections and corresponding immunohistochemical staining sections **(B–D)** Expression of pro-inflammatory cytokines in all treatment groups.

The MIA model is the most successful and frequent OA model, and its pathological characteristics are very similar to human OA (Pomonis et al., 2005; Liu et al., 2011; Malfait et al., 2013). Because of its good *in vitro* experimental results, we established an OA animal model to evaluate the therapeutic effect of NP_DXM/PPLA_. Three days after the successful formation of artificial OA, 40 mice were divided into 4 groups: NS group, NP_PPLA_ group, DXM group, and NP_DXM/PPLA_. OA mice were injected with drugs of each group at a weight of kg every 4 days. Mice without OA induction were set as a control group. The degree of articular cartilage destruction, extracellular matrix loss, and changes in inflammatory cytokines (IL-1β, IL-6, and TNF-α) was measured after treatment. As shown in Figure 7A, H&E staining sections of chondrocytes in the normal group showed neat chondrocytes and no synovial hyperplasia around the joint. In comparison to the control group, the NP_DXM/PPLA_ treatment group had less inflammatory cell infiltration and only minor chondrocytes degeneration, whereas the normal saline treatment group had more obvious chondrocyte destruction and severe synovial hyperplasia with a high number of inflammatory cell infiltration.

Proinflammatory cytokines expressed by macrophages have been shown to play an important role in the early stages of OA and in promoting disease progression (Blanco et al., 2011; Huang and Kraus, 2016). Inhibiting the release of proinflammatory cytokines in OA tissue therefore can effectively reduce the degree of cartilage damage (Agarwal et al., 2016; Utomo et al., 2016, 2019; Chevalier and Eymard, 2019). To further investigate the anti-inflammatory effect of the NP_DXM/PPLA_ group, the expression of proinflammatory cytokines including IL-1β, IL-6, and TNF-α in mouse knee osteoarthritis specimens was determined by an ELISA. The expressions of proinflammatory cytokines IL-1β, IL-6, and TNF-α were lower in the NP_DXM/PPLA_ group compared to the saline group, as shown in Figures 7B–D. The expression of proinflammatory cytokines in the NP_DXM/PPLA_ treatment group was also decreased.

**FIGURE 8:**
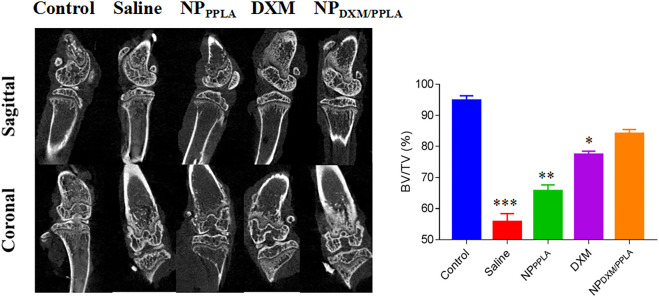
The efficacies of NS, NP_PPLA_, DXM and NP_DXM/PPLA_ on OA were observed by Micro CT and analyzed the bone volume fraction (BV/TV) in subchondral region.

Next, we evaluated the knee bone structure of OA mice by Micro CT. The comparison of bone volume fraction (BV/TV) in the subchondral region of the knee in mice, as shown in Figure 8, revealed that the bone mass in the saline group was approximately 55%, while that in the NP_DXM/PPLA_ group was 90%. The bone mass of the knee subchondral bone in mice treated with normal saline was importantly lesser than that in mice treated with NP_DXM/PPLA_, whereas the bone mass of the knee in mice treated with NP_DXM/PPLA_ was similar to that in healthy mice. Taken together, all these outcomes show that NP_DXM/PPLA_ is promising for the treatment of OA.

## 4 Conclusion

In this study, we synthesized NP_DXM/PPLA_ through polymerization and hydrophilic interactions. *In vitro* characterization affirmed that NP_DXM/PPLA_ can carry and release DXM effectively. It also revealed good stability under physiological conditions. FCA and CLSM confirmed that NP_DXM/PPLA_ can be activated by macrophage endocytosis and release. Finally, synovial inflammation was reduced in the OA mice model, and cartilage destruction and bone loss were inhibited, which was closer to the knee joint of the control mice. As a result, NP_DXM/PPLA_ can be used to treat osteoarthritis and is a potential vector material for clinical use. At present, the research on drug delivery, targeting, controlled release and other aspects is the forefront of drug delivery systems. In this study, drug delivery nanoparticles were prepared by simple and easy methods, achieving a more stable drug controlled release, which has a certain anti osteoarthritis effect, providing a certain reference for the development of more efficient drug delivery systems in the future.

## Data Availability

The original contributions presented in the study are included in the article/supplementary materials, further inquiries can be directed to the corresponding author.
